# Antiretrovirals for low income countries: an analysis of the commercial viability of a highly competitive market

**DOI:** 10.1186/1744-8603-9-6

**Published:** 2013-02-15

**Authors:** Olive N Nakakeeto, Brian V Elliott

**Affiliations:** 1 Independent health consultant, 387 Vi de Sales, F-01710, Thoiry, France; 2 Independent pharmaceutical industry consultant, 18 Deerpark Lawn Castleknock, Dublin 15, Ireland

**Keywords:** Antiretroviral drugs, HIV, AIDS, Production cost, Market analysis

## Abstract

**Background:**

The price of antiretroviral drugs (ARVs) in low income countries declined steadily in recent years. This raises concerns about the commercial viability of the market of ARVs in low income countries.

**Methods:**

Using 2 costing scenarios, we modeled the production cost of the most commonly used ARVs in low income countries in 2010 and 2012, and assessed whether, at the median price paid by low income countries, their manufacturers would still make profits. By interviews we consulted 11 generic manufacturers on the current state of the ARV market, and on what would be required to ensure their continued commitment to supply ARVs to low income countries.

**Results:**

Using the lowest prices for active pharmaceutical ingredients (API) quoted to WHO, and applying published assumptions about the production cost of ARVs, our baseline estimate was that Indian generic manufacturers would have made profits on only 1 out of 13 formulations of ARVs in both 2010 and 2012, and publicly owned manufacturers would have made profits on 5 and 3 out of 13 formulations in 2010 and 2012, respectively. We needed to assume a 20% and a 40% lower API cost for our model to predict that publicly owned and Indian manufacturers, respectively, would make profits on the sale of the majority of their ARVs. Between 2010 and 2012, we estimate that - across the ARV portfolio - the gross profit on sales of ARVs to low income countries decreased with between 6% and 7% of their sales price. Generic manufacturers consider that current prices are unsustainable. They suggested amendments to the tender procedures, simplified regulatory procedures, improved forecasting, and simplification of the ARV guidelines as critical improvements to maintain a viable ARV market.

**Conclusions:**

While recent price decreases indicate that there is still space for price reduction, our estimate that gross profit margin on sales decreased by 6 to 7% between 2010 and 2012 lends credibility to assertions by generic manufacturers that the ARV market in low income countries is under considerable price pressure. This might create problems for the quality and/or the continued supply of ARVs to low income countries.

## Background

At the end of 2011, more than 8 million people received antiretroviral treatment. This represents 54% of the estimated 14.8 million people eligible for treatment [1], and represents major progress towards the target of treating 15 million people by 2015, to which the global community committed in the UN General Assembly in 2011 [2]. The dramatic scale up of antiretroviral treatment was made possible thanks to the commitments and action of countries and the donor community, but also because of a steady decrease in the cost of the drugs they use. When, ten years ago, it cost close to 10.000 $US for a year’s treatment, the median cost of drugs used in first line treatment in low income countries decreased to between 57 $US (for the adult fixed dose combination of stavudine, lamivudine and nevirapine) and 186 $US (for the adult fixed-dose combination of tenofovir, emtricitabine and efavirenz) per year in 2012 [3].

While, according to UNITAID, the ARV market in low income countries increased in value from $US 188 million ARV in 2005 to $US 835 million in 2010, and the number of manufacturers supplying World Health Organisation, (WHO)-prequalified quality assured products increased from 17 in 2005 to 26 in 2010 [4], generic manufacturers increasingly assert that the current low prices and trends to further reduce them seriously threaten market sustainability [5].

We decided to verify their claims that current price levels are unsustainable by analyzing their production costs and profit margins, to interview them to document their perception of what they consider are key constraints in the low income country market for ARVs, and to identify which interventions might be considered to overcome them. This paper presents our analysis of the ARV production costs and profits made from their sale in low income markets, and reports on the outcome of the interviews with the manufacturers.

## Methodology

### Pricing analysis

From WHO’s 2010 survey on the use of ARVs we selected all WHO-recommended regimens which in 2010 were used by more than 95% of 1^st^ line patients, and the 2 most frequently used 2^nd^ line regimens [6]. From the WHO Global Price Reporting Mechanism [3], we identified which formulations (and dosages) were most frequently used to administer those regimens. We then calculated the quantity and the cost of APIs needed to treat one patient for one year (365 days) with each of those formulations, using the lowest API price quoted by their manufacturers in the 2010 and 2012 WHO surveys on the price and production capacity of APIs for ARVs [7,8], and calculated their total production cost, using 2 scenarios.

In the first scenario, “Indian producers”, we modeled the total production cost for privately owned Indian generic manufacturers. From the 2010 annual reports of publicly listed Indian generic ARV manufacturers (Cipla Ltd [9], Ranbaxy Laboratories [10], Strides Arcolab Ltd [11] and Aurobindo Pharma Ltd [12]) we extracted the expenditures incurred in the production of their medicines (material cost, employee cost, operating and other expenditures, research and development expenses, interest, depreciation and amortization of goodwill), and added the value of each expenditure category to obtain their value across the 4 companies (Table 1). Across the 4 companies, the cost of materials - including API, excipients and packaging - was 56.1% of the total production cost. As the cost of excipients and packaging materials was not reported separately in the annual reports, we assumed that their cost would be the same as that in Brazil, which we calculated to be 3.21 $US for every 365 tablets produced ,from a paper by Eloan Pinheiro et al. [13], as described below. As beyond the cost of materials, the total production cost contained on average 43.9% costs to cover employee costs, operating and other costs, research and development cost, interest, and depreciation and amortization of goodwill, we added 78.3% (43.9/56.1) to the cost of materials (APIs, excipients and packaging) to calculate the total production cost.

**Table 1:** Summary of the 2010 annual reports of publicly traded Indian ARV manufacturers (*)

	**CIPLA**		Ranbaxy (**)		**Strides Arcolab**		**Aurobindo**		**Total of 4 companies**	
**Gross sales of goods (minus excise duty)**	**61,351**		**56,721**		**16,958**		**39,979**		**175,009**	
**Expenditure**		% of expenditures		% of expenditures		% of expenditures		% of expenditures		% of expenditures
Material cost	28,604	54.1	21,709	46.2	8,508	52.4	23,286	76.9	82,107	56.1
Employee cost	4,642	8.8	7,761	16.5	2,249	13.8	3,036	10.0	17,688	12.1
Operating and other expenses	14,641	27.7	14,712	31.3	3,388	20.8	6,716	22.2	39,457	27.0
Research & Development Expenses	2,598	4.9							2,598	1.7
Interest	50	0.1	542	1.1	**1,**467	9.0	505	1.6	2,564	1.8
Depreciation and Amortization of goodwill	2,289	4.3	2,284	4.8	638	3.9			4,611	3.1
Total	**52,824**	100	**47,018**	100	**16,250**	100	**30,291**	100	**146,383**	100
		% of sales		% of sales		% of sales		% of sales		% of sales
**Gross Profit before taxes**	**8,527**	**13.9**	**9,703**	**17.1**	**1,858**	**11.0**	**9,688**	**24.2**	**28,626**	**16.4**

(*) Amounts in million Rupees.

(**) The expenditures of Ranbaxy exclude an extraordinary charge to account for future liabilities.

In the second scenario “Publicly owned producer”, we modeled the total production cost for a publicly owned production facility, using data from Brazil [13]. The cost of the materials was calculated as the sum of the cost of the API used, plus the cost of the excipients and packaging. The cost of excipients and packaging was calculated as 3.21 $US/365 tablets produced, summing up the cost of excipients and packaging for each of the 7 formulations included in Table 1 from the paper on the cost structure of the production of ARVs from Brazil [13], and dividing it by 20 - the total number of tablets included in a defined daily dose of each of the seven formulations combined. To obtain the total production cost, the cost of labor and equipment, quality control and transport, and indirect costs needed to be added. Together those amounted to 17.4%, or 468 $US, of the total sales price of 2691 $US of the 7 formulations included in the same table. Dividing this amount by 20 - the total number of tablets included in a defined daily dose of each of the seven formulations combined Ó yielded 23.4 $US as the estimate of their average contribution to the total production cost for 365 tablets.

Table 2 illustrates how the scenarios were used to calculate the total production cost of one person-year of lamivudine 150 mg plus zidovudine 300 mg combination tablets.

**Table 2:** Production cost and profits made from lamivudine 150 mg plus zidovudine 300 mg tablets

**Materials cost**	**Zidovudine**	**Lamivudine**
Daily dose (mg)	600	300
Amount of API per person-year in kg (daily dose in mg X 365 days/1.000.000)	0.219	0.1095
Cost of API (per kg) (lowest quote in the 2010 WHO survey)	315	150
Cost of API per person-year (Amount of API per person-year in kg X cost of API per kg)	69.0	16.4
Cost of API per person-year of both APIs combined	85.4	
Number of tablets/day required to administer a daily dose of zidovudine plus lamivudine	2	
Cost of excipients and packaging (3.21 per 365 tablets X number of tablets/day X 365 days)	6.4	
**Subtotal materials cost per person-year**	91.8	
**Non-materials cost per person-year (according to both scenarios)**	**Indian manufacturer**	**Publicly owned manufacturer**
Indian manufacturer: 78.3% of materials cost	71.9	-
Publicly owned manufacturer: 23.4 X number of tablets/day	-	46.8
**Total production cost per person-year**		
Materials cost PLUS non-materials cost	163.7	138.6
**Median price paid by low income countries per person- year in 2010**	101.4	
**Profit or loss per person-year at API cost quoted to WHO in 2010**	−62.3	−37.2
**Profit or loss per person-year when API cost is:**		
20% less than quoted to WHO	−31.9	−20.1
40% less than quoted to WHO	−1.4	−3.1
60% less than quoted to WHO	29.0	14.0

(amounts in $US).

Following calculation of the total production costs of each formulation, we calculated whether, at the median price paid for them by low income countries in 2010 and 2012 (taken from the WHO Global Price Reporting Mechanism [3]), and under both scenarios, the companies producing them would have made profits or losses, and we assessed whether our assessment of their profits and losses would be materially altered if we varied the cost of the APIs, in a univariate sensitivity analysis. Finally, we assessed the evolution of their margins between 2010 and 2012, comparing arithmetic mean profit margins across the ARVs considered in the sensitivity analysis.

### Interviews with manufacturers of generic antiretroviral drugs

We consulted 11 manufacturers producing ARVs, as finished formulations or as APIs (Aspen Pharmacare, Aurobindo Pharma Ltd, Cipla Ltd, Desano, Emcure, Hetero, Laurus Labs, Mylan/Matrix, Ranbaxy Laboratories, Strides Arcolab Ltd and UCL) on the current state of the ARV market, and steps which they consider necessary to ensure their continued commitment to supplying ARVs in the future.

## Results

### Pricing analysis

According to the 2010 WHO survey on ARV use [6], the most frequently used first line regimens (with % of patients on first line therapy using them) were lamivudine plus stavudine plus nevirapine (27.7%), lamivudine plus stavudine plus efavirenz (14%), lamivudine plus zidovudine plus nevirapine (used by 26.8%), lamivudine plus zidovudine plus efavirenz (11.4%), lamivudine plus tenofovir plus efavirenz (10.6%), emtricitabine plus tenofovir plus efavirenz (3.5%), and lamivudine plus tenofovir plus nevirapine (2.7%). The two most frequently used second line regimens (with % of patients using second line therapy using them) were lamivudine plus tenofovir plus lopinavir (boosted with ritonavir) (27.1%), and zidovudine plus didanosine plus lopinavir (boosted with ritonavir) (25%).

Taking into account the relative volume of different formulations sold to low income countries reported in the WHO Global Price Reporting mechanism, we identified the 13 formulations listed in Table 3 as the most frequently used formulations to administer those regimens. Table 3 lists, under the “Indian manufacturer” and “Publicly owned producer” scenarios, the median price paid by low income countries (from [3]), the lowest price of the APIs quoted to WHO in 2010 and 2012 (from [7] and [8]), our calculation of the total production cost, and our estimate of profit or loss generated by the sale of one person-year of each formulation, at 2010 and 2012 median prices. In addition the effect of lowering the price of the active pharmaceutical ingredients by 20%, 40% and 60% is shown.

**Table 3:** Profits and losses made from the sale of antiretroviral drugs to low income countries

**Formulation and defined daily dose**	**2010**							**2012**						
	**Me-dian price**	**Lowest API cost quoted to WHO**	**Total production cost**	**Profit or loss at API cost**				**Me-dian price**	**Lowest API cost quoted to WHO**	**Total production cost**	**Profit or loss at API cost**			
				**quoted to WHO**	**20%**	**40%**	**60%**				**quoted to WHO**	**20%**	**40%**	**60%**
					**less**	**less**	**less**					**less**	**less**	**less**
**1) Scenario “Indian manufacturers”**														
**Three drug combination tablets**														
[lamivudine 150 mg + stavudine 30 mg + nevirapine 200 mg], b.i.d	63	37	78	−15	−2	11	25	57	39	81	−28	−10	4	18
[lamivudine 150 mg + zidovudine 300 mg + nevirapine 200 mg] b.i.d.	131	87	166	−4	−4	27	58	119	90	171	−53	−21	11	43
[lamivudine 300 mg + tenofovir 300 mg + efavirenz 600 mg] q.d.	192	126	230	−38	7	52	97	162	102	187	−25	11	47	84
[emtricitabine 200 mg + tenofovir 300 mg + efavirenz 600 mg] q.d.	242	136	243	−1	47	94	142	186	126	224	−38	6	50	93
**Two drug combination tablets**														
[lamivudine 150 mg + stavudine 30 mg] b.i.d.	39	24	54	−15	−7	2	10	36	24	55	−19	−10	−1	7
[lamivudine 150 mg + zidovudine 300 mg] b.i.d.	101	73	142	−41	−23	3	30	94	75	145	−51	−25	2	29
[lamivudine 300 mg + tenofovir 300 mg] q.d.	109	71	133	−24	2	27	52	65	58	109	−44	−23	−3	18
[emtricitabine 200 mg + tenofovir 300 mg ] q.d.	143	78	145	−2	26	54	82	87	73	136	−49	−23	3	29
[lopinavir 200 mg + ritonavir 50 mg], 4 tablets/day	433	405	745	−313	−169	−24	121	359	314	583	−223	−112	0	112
**Single drug formulations**														
[efavirenz 600 mg] q.d.	56	55	103	−47	−28	−8	11	46	44	84	−37	−22	−6	9
[nevirapine 200 mg] b.i.d.	31	13	35	−4	1	6	10	30	16	40	−10	−5	1	7
[zidovudine 300 mg] b.i.d	88	57	113	−25	−5	15	36	82	59	117	−35	−14	8	29
[didanosine 250 mg] b.i.d	159	52	105	54	73	92	110	165	55	109	55	75	94	114
**Summary**														
Column total	1787	1214	2292	−475	−82	351	784	1488	1074	2041	−557	−173	210	592
Arithmetic mean profit margin (%)				−27	−5	20	44				−37	−12	14	40
Decrease in profit margin between 2010 and 2012 (%)											11	7	6	4
Average decrease in profit margin across sensitivity analysis outcomes (%)											7			
**2) Scenario “Publicly owned producer”**														
**Three drug combination tablets**														
[lamivudine 150 mg + stavudine 30 mg + nevirapine 200 mg], b.i.d	63	37	91	−28	−21	−13	−6	57	38.8	92	−35	−27	−20	−12
[lamivudine 150 mg + zidovudine 300 mg + nevirapine 200 mg] b.i.d.	131	87	140	−9	8	26	43	119	89.6	143	−24	−6	12	30
[lamivudine 300 mg + tenofovir 300 mg + efavirenz 600 mg] q.d.	192	126	153	40	65	90	115	162	102	128	34	54	74	95
[emtricitabine 200 mg + tenofovir 300 mg + efavirenz 600 mg] q.d.	242	136	159	83	109	136	162	186	126	149	37	62	86	111
**Two drug combination tablets**														
[lamivudine 150 mg + stavudine 30 mg] b.i.d.	39	24	77	−38	−33	−29	−24	36	24	77	−42	−37	−32	−27
[lamivudine 150 mg + zidovudine 300 mg] b.i.d.	101	73	127	−25	−18	−3	11	94	75	128	−34	−19	−4	11
[lamivudine 300 mg + tenofovir 300 mg] q.d.	109	71	98	11	25	40	54	65	58	85	−19	−8	4	15
[emtricitabine 200 mg + tenofovir 300 mg] q.d.	143	78	105	38	54	70	85	87	73	99	−13	2	16	31
[lopinavir 200 mg + ritonavir 50 mg], 4 tablets/day	433	405	512	−79	2	83	164	359	314	374	−14	48	111	174
**Single drug formulations**														
[efavirenz 600 mg] q.d.	56	55	81	−25	−14	−3	7	46	44	70	−24	−15	−6	2
[nevirapine 200 mg] b.i.d.	31	13	66	−35	−33	−30	−27	30	16	69	−40	−36	−33	−30
[zidovudine 300 mg] b.i.d	88	57	110	−22	−11	0	12	82	59	112	−30	−18	−6	5
[didanosine 250 mg] b.i.d	159	52	106	54	64	75	85	165	55	108	57	67	78	89
**Summary**														
Column total	1787	1214	1825	−35	197	442	681	1488	1074	1634	−147	67	281	494
Arithmetic mean profit margin (%)				−2	11	25	38				−10	4	19	33
Decrease in profit margin between 2010 and 2012 (%)											8	7	6	5
Average decrease in profit margin across sensitivity analysis outcomes (%)											6			

(amounts in whole $US per person-year).

In the scenario “Indian manufacturers”, at an API price equal to their lowest quoted price to WHO, only the sale of didanosine 250 mg tablets is predicted to generate profit, in both 2010 and 2012. One needs to assume a 20% lower API price for 6 formulations to generate profit in 2010, but at this reduced API cost, only 3 formulations are predicted to generate profit in 2012. In 2010 and 2012, 11 and 10 formulations, respectively, are predicted to generate profit at a 40% decreased API price. At a 60% lower API cost all formulations are predicted to generate profits, in both years. Considering the profit (or loss) margins made on aggregate sales price of the 13 formulations combined, one needs to assume an API price decrease of almost 40% to generate a gross profit margin of 20% in 2010. By 2012, the gross profit margin generated when the API cost is reduced by 40% is seen to have decreased to 14%, a 6% loss in 2 years. Across the different levels of API price, there is a decrease in profit margin from 2010 to 2012 of 7%.

In the scenario “Publicly owned producer”, 3 out of the 13 formulations are predicted to return profits in 2010 and 2012, at the API prices quoted to WHO. One needs to assume a 20% lower than quoted API cost for 7 and 5 formulations to return a profit, and at a 40% lower API cost 8 and 7 formulations become profitable, in 2010 and 2012, respectively. At a 60% lower API cost, 10 formulations are predicted to generate profits in both years. Considering the profit (or loss) made on aggregate sales price of the 13 formulations combined, one needs to assume an API price decrease of almost 20% to generate a gross profit margin of 11% in 2010, across the formulations considered. By 2012, the aggregate gross profit margin generated when the API cost is reduced 20% is seen to have decreased to 4%, a 7% loss in 2 years. Across the different levels of API price, there is a decrease in profit margin from 2010 to 2012 of 6%.

### Interviews with manufacturers of generic antiretroviral drugs

In their interviews the manufacturers assured us that there is still a strong commitment to supply appropriate treatments to HIV/AIDS patients in low income countries, as many acknowledged and pledged to continue embracing their social obligations. On the other hand, most manufacturers interviewed stated that they would “Only stay in the ARV market if the business is profitable in the longer term”.

The most frequently mentioned constraints included rapidly decreasing profitability of the low income country ARV market, difficult market entry, and limited access to reliable forecasting information.

The competitive advantage of vertically integrated companies (producing both API’s and finished formulations), who control API prices and can base their quoted prices for formulations on end- to- end margins was characterized by several interviewees as unfair, as it eliminates companies which cannot control API prices from receiving tender awards. In addition, non-Indian manufacturers mentioned export subsidies offered by the Indian Government to Indian manufacturers and exporters as constraining their ability to compete.

Elaborating on the rapidly decreasing profitability, they stated that the prices for first line ARV drugs “are at the bottom”; with average portfolio gross margins at 5 to 8 percent. They also mentioned that, while initially profit margins on generic second line and new first line products might reach 20 to 25 percent, they eroded drastically between 2010 and 2012.

According to the manufacturers, the current low prices for generic products discourage new market entrants, placing reliance on a small number of companies to cater for greatly increased demand in the coming years, while discouraging the very same companies to maintain a strong presence. The companies interviewed estimated that the industry has adequate formulation capacity, but that, with new product opportunities arising in other therapeutic areas, it is increasingly difficult to keep production capacity allocated to low margin ARVs. While currently assigned API capacity would need to increase by 40% to treat 20 million patients, even maintaining current production levels was mentioned to be problematic at current prices, in particular in India, where current interest rates were reported to stand at 13 to 17% for major investment loans. One manufacturer stated that an API plant normally has a production life span of 7Ó8 years, and might cost 32 to 35 million $US to replace Ó an estimate given without information on its production capacity, but corroborated subsequently as a “typical figure” by 2 additional manufactures. It was mentioned that in the last 4 to 5 years there were no new API market entrants, and that current API production comes from plants which are nearing the end of their normal production life span.

Elaborating on market entry difficulties, they pointed out that Ó with new medicines taking between 3 to 5 years after FDA or EMEA approval to launch in developing countries, as it requires inclusion in WHO treatment guidelines, prequalification and multiple registrations - the attractiveness of the market is further curtailed. Moreover, slow and costly drug registration processes were characterized as unsustainable for low volume products or for countries with small numbers of patients.

The remedial actions suggested by the manufacturers include, first, to alleviate the effects of the price pressure under which they operate. Abolition of the lowest tender price rule and sharing the market ( e.g., the lowest bidder to supply 60%, the second, third and fourth lowest bidders to supply 20%, 10% and 10% respectively *at the price of the lowest bidder)* would not increase the cost to the buyer, but would enable companies to manage their stocks and production of APIs and formulations with less risk, and avoid distress sales when drugs are approaching their shipping deadlines (well in excess of 1 year before they expire). In addition, there should be no obligation to bid on all items in a tender Ó as most companies do not produce all products required. Finally, it would help if the clients paid on time: the example of PEPFAR and its Supply Chain Management System, who pays within 45 days, which in turn enables formulators to pay their API suppliers in 90 days, and generates positive cash flow, was cited as a positive example and practice to be emulated. Country tenders, often marred by late payments, irregular off takes and cash flow problems, were cited as increasingly problematic.

Faster registration processes, including regional regulatory harmonization and fast track registration of prequalified or stringent regulatory approved ARVs were called for. In addition, there were calls to discourage inspections by national drug regulatory authorities when facilities have been inspected by a stringent regulatory authority or WHO. Several interviewees also articulated the need to further simplify antiretroviral treatment guidelines, as the number of drug combinations in present use was perceived to be excessive, requiring too many registrations, with serious time and cost implications.

Reliable demand forecasts and sharing of information on future therapeutic developments between technical agencies and pharmaceutical companies was also identified as a need to ensure that the development of products fits the ARV recommendations. The need to harmonize forecasts by WHO and the Clinton Health Access Initiative was identified as an area which must improve in the short term.

Finally, earlier access for generic manufacturers to new drugs and new formulations from innovators and launching new products in low income countries coincidental with developed markets was cited by many as an important priority.

## Discussion

In the annual meeting of WHO and UNAIDS with ARV producers in October 2011 [5], manufacturers stated that for several ARVs the gross margin is now less than 10% - typically 5 to 8%. They pointed out that, as new opportunities arise (they reported that in 2012 alone 67 medicines will come off patent in the USA) it is increasingly difficult to convince manufacturers to expand or even to maintain existing ARV production capacity. As competitive markets do require a diverse offer, the increasingly vocal statements by the producers sparked off several assessments by the industry, and not-for-profit organizations, such as UNITAID [4] and the Global Fund.

Our own attempt to analyse whether producing ARVs for low income countries was still profitable was hampered by lack of transparency about the production costs of ARVs. Key uncertainties concern the cost of the APIs, and the structure of the manufacturing costs of the generic producers.

The first major uncertainty is the cost of APIs to ARV producers. We used the lowest price quoted for APIs from consecutive surveys by WHO, in spite of the fact that those prices were consistently on average 35% lower than the prices quoted for the same APIs in the World Trade Organization, WTO Market News Service [14]. We had to conclude, from our sensitivity analysis, and taking into account that the companies still publicly stated that they continued to make Ó albeit modest Ó profits, that even those lowest quotes to WHO are overestimated. This conclusion is also supported by a case documented in our interviews in which a vertically integrated API manufacturer marked up the price of one of its API to a formulator with at least 35% to ensure its own competitiveness in a 2012 tender.

A second uncertainly is the cost structure of ARV production in the leading ARV producing companies. While, on average, the publicly quoted Indian ARV manufacturers reported 43.9% expenditure on non-material costs, this is much higher than the 17.4% non-material costs reported from Brazilian manufacturers [13]. As non-materials cost likely becomes less, as a proportion of the total production cost, when volumes increase, as is the case with antiretrovirals, we therefore likely overestimated their magnitude in our assessment of the production cost of the Indian manufacturers. Also hinting in this direction was a statement from an Indian manufacturer that costs across his first line portfolio were API 80%, excipients 15%, and packaging 1%, leaving a 4% gross margin. His non-materials cost was apparently so low as to have been forgotten.

A final limitation of our costing analysis is that we added a fixed amount to account for excipients and packaging costs, to the cost of API, for every 365 tablets produced. This is clearly suboptimal, as different formulations come with different excipients, and their cost will change over time. While this cost was derived from published data from Brazil [13], it might be overestimated for Indian producers, which operate on a much larger scale than their Brazilian counterparts.

When considering how worried one should be about the commercial viability of the ARV market in low income countries, there are conflicting signals.

On one hand, there are signals that there is still scope for price reduction, in particular for the newer formulation products containing tenofovir. In December 2012, the government of South Africa announced that it has secured access to lower prices for several antiretroviral drugs, including the fixed dose combination of emtricitabine, tenofovir, and efavirenz for a substantially reduced price Ó quoted as 89.37 Rand per patient per month, equivalent to 120 US$ per year of treatment [15]. It is hard to imagine that the 3 companies (Aspen Pharmacare, Cipla Medpro and Mylan Pharmaceuticals) which agreed to supply this formulation would do so at a loss.

On the other hand, we have converging statements that the gross profit margins on the first line portfolio have decreased to between 5% and 8%, considerably less than the average gross profit margin of 16% reported by publicly quoted Indian ARV manufacturing companies listed in Table 1, and less than the gross 14% margin reported for a generic manufacturer in Brazil [13]. Our assessment of the evolution of their profit margins suggests that they might have lost 6 to 7% of their gross profit margin on sales between 2010 and 2012 is in keeping with their assertions. This would explain why their HIV product managers face challenges when pleading with their senior management to expand ARV production.

A second distress signal is that manufacturers are beginning to drop out of more challenging markets. CIPLA, the Indian company which started supplying affordable ARVs to low income countries in 2002, announced in July 2012 that, after shifting its export portfolio away from the tender ARV business, will cut down exposure to anti-HIV drugs, and shift it to high-margin, complex products such as inhalers [16]. Also, while a denial was posted in e-drug, in early 2012, the Zimbabwean manufacturer Varichem was reported to have stopped producing ARVs, and stated that it had not supplied any ARVs to Zimbabwe since November 2010, citing lack of local support [17,18].

Some suggestions to protect the future development of the market for antiretroviral drugs in low income countries have been identified in assessments by UNITAID and Médecins Sans Frontières (MSF). The UNITAID Medicines landscape report identified, among others, the need to secure API supply for antiretroviral medicines, and suggested to focus on developing market intelligence on API markets to identify suppliers, production capacity, cost structures, intellectual property issues, and relationships between API suppliers and finished product manufacturers, and to support upstream API production expansion where needed through incentives [4]. Concurring with the manufacturers we interviewed, their report further suggests generating and sharing regular market forecasts at API and final product levels, to improve transparency and minimize market uncertainty for developers and manufacturers [4]. The 2012 “Untangling the Web” report by Médecins Sans Frontières includes a strong focus on intellectual property as a tool to sustain competitive markets, and, in pleading for further price decreases, recognizes the need to maintain a competitive environment for which the continued commitment of generic manufacturers from India remains critical [19]. There is also agreement with the manufacturers , strongly articulated by WHO and UNAIDS in Treatment 2.0 initiative, that simplification of the treatment guidelines is needed to increase access to antiretroviral therapy [20].

However, even if there remains uncertainty about whether the prices in the ARV market for low income countries have really bottomed out, increasing access also requires that the views the companies which compete with one another to supply the market be given serious consideration. This requires attention to how the business is concluded, how fast they are paid, which regulatory hurdles need to be overcome, and what intelligence can be shared to create the global public good that access to treatment for HIV represents.

The opinions and actions of organizations such as WHO, UNAIDS and the Global Fund in changing the ways tenders are conducted, in pharmaceutical regulation, and in forecasting will be critical in achieving the goal Ó 15 million people on ARV treatment by 2015 Ó which they helped formulate. However, it would help the manufacturers too if they could be a bit more forthcoming with information that would allow a better appraisal of whether their prices are fair and viable.

## Abbreviations

API: Active pharmaceutical ingredients;ARV: Antiretroviral drugs;EMEA: European Medicine Agency;FDA: Food and Drug Administration;PEPFAR: President's Emergency Plan for AIDS Relief;UNAIDS: United Nations Program on HIV/AIDS;WHO: World Health Organisation;WTO: World Trade Organization

## Competing interests

ONN declares that she has no competing interests. BVE has received funding from Johnson & Johnson Inc. to cover the cost of the interviews and their reporting, included in this manuscript. He has worked in addition, as a consultant with several other research-based pharmaceutical companies producing antiretroviral drugs.

## Authors ’ contributions

ONN conceptualized and carried out the price analysis. BVE conducted the interviews with the pharmaceutical manufacturers. They wrote the corresponding sections of the manuscript and jointly wrote the discussion, conclusions and background sections. Both authors read and approved the final manuscript.
